# The protective effects of ligustrazine on ischemic stroke: a systematic review and meta-analysis of preclinical evidence and possible mechanisms

**DOI:** 10.3389/fphar.2024.1373663

**Published:** 2024-03-13

**Authors:** Ziming Wang, Zihong Wu, Yifan Miao, Aohan Hao, Hao Chen, Shuang Zhao, Min Luo, Shihan Guo, Yingming Liu, Yun Lu

**Affiliations:** ^1^ Hospital of Chengdu University of Traditional Chinese Medicine, Chengdu, China; ^2^ Hubei University of Chinese Medicine, Wuhan, China

**Keywords:** ligustrazine, ischemic stroke, inflammation, oxidative stress, apoptosis, meta-analysis

## Abstract

**Introduction:** The objective of this study is to systematically evaluate the effect of ligustrazine on animal models of ischemic stroke and investigate its mechanism of action.

**Materials and Methods:** The intervention of ligustrazine in ischemic diseases research on stroke model animals was searched in the Chinese National Knowledge Infrastructure (CNKI), Wanfang Database (Wanfang), VIP Database (VIP), Chinese Biomedical Literature Database (CBM), Cochrane Library, PubMed, Web of Science, and Embase databases. The quality of the included literature was evaluated using the Cochrane risk of bias tool. The evaluation included measures such as neurological deficit score (NDS), percentage of cerebral infarction volume, brain water content, inflammation-related factors, oxidative stress-related indicators, apoptosis indicators (caspase-3), and blood-brain barrier (BBB) permeability (Claudin-5).

**Results:** A total of 32 studies were included in the analysis. The results indicated that ligustrazine significantly improved the neurological function scores of ischemic stroke animals compared to the control group (SMD = −1.84, 95% CI −2.14 to −1.55, *P* < 0.00001). It also reduced the percentage of cerebral infarction (SMD = −2.97, 95% CI −3.58 to −2.36, *P* < 0.00001) and brain water content (SMD = −2.37, 95% CI −3.63 to −1.12, *P* = 0.0002). In addition, ligustrazine can significantly improve various inflammatory factors such as TNF-α (SMD = −7.53, 95% CI −11.34 to −3.72, *P* = 0.0001), IL-1β (SMD = −2.65, 95% CI −3.87 to −1.44, *P* < 0.0001), and IL-6 (SMD = −5.55, 95% CI −9.32 to −1.78, *P* = 0.004). It also positively affects oxidative stress-related indicators including SOD (SMD = 4.60, 95% CI 2.10 to 7.10, *P* = 0.0003), NOS (SMD = −1.52, 95% CI −2.98 to −0.06, *P* = 0.04), MDA (SMD = −5.31, 95% CI −8.48 to −2.14, *P* = 0.001), and NO (SMD = −5.33, 95% CI −8.82 to −1.84, *P* = 0.003). Furthermore, it shows positive effects on the apoptosis indicator caspase-3 (SMD = −5.21, 95% CI −7.47 to −2.94, *P* < 0.00001) and the expression level of the sex-related protein Claudin-5, which influences BBB permeability (SMD = 7.38, 95% CI 3.95 to 10.82, *P* < 0.0001).

**Conclusion:** Ligustrazine has been shown to have a protective effect in animal models of cerebral ischemic injury. Its mechanism of action is believed to be associated with the reduction of inflammation and oxidative stress, the inhibition of apoptosis, and the repair of BBB permeability. However, further high-quality animal experiments are required to validate these findings.

## 1 Background

Stroke is the second leading cause of death and the third leading cause of disability in the world (Lozano et al., 2012; Murray et al., 2012). In 2016, there were 13.7 million new stroke events worldwide, with 87% of them being ischemic strokes. During the same year, 2.7 million people died from stroke. Cerebral ischemic stroke (CIS) imposes a significant health and economic burden on the world, particularly on low- and middle-income countries (Collaborators, 2019; Saini et al., 2021). CIS is typically caused by embolism or thrombotic artery occlusion, resulting in reduced cerebral blood flow and various forms of brain damage, including tissue and structural damage, as well as neuronal death and defects (Xing and Bai, 2020; Zhao et al., 2022). Recombinant tissue plasminogen activator (tPA) is currently the only CIS therapy approved by the U.S. Food and Drug Administration. However, this therapy has a limited time window of 4.5 h and carries the potential risk of hemorrhagic transformation, resulting in only 10% of stroke patients benefiting from it (Bansal et al., 2013; Tao et al., 2020). Recent advancements in the research of stroke neuroprotective drugs have identified promising candidates such as human urinary kininogenase (HUK), statins, edaravone, 3K3A-activating protein C (APC), and vinpocetine. These drugs target multiple pathophysiological pathways involved in stroke-related brain injury, with a primary focus on inflammation and oxidative stress. (Paul and Candelario-Jalil, 2021). While these studies provide optimism for CIS treatment, further research is needed to fully address the complexities of this condition. Moreover, there is an urgent need to enhance the current treatment’s effectiveness in addressing motor dysfunction and neurological damage caused by CIS. Therefore, it is crucial to explore new complementary and alternative therapies to overcome the limitations of existing treatments in stroke management.

**FIGURE 1:**
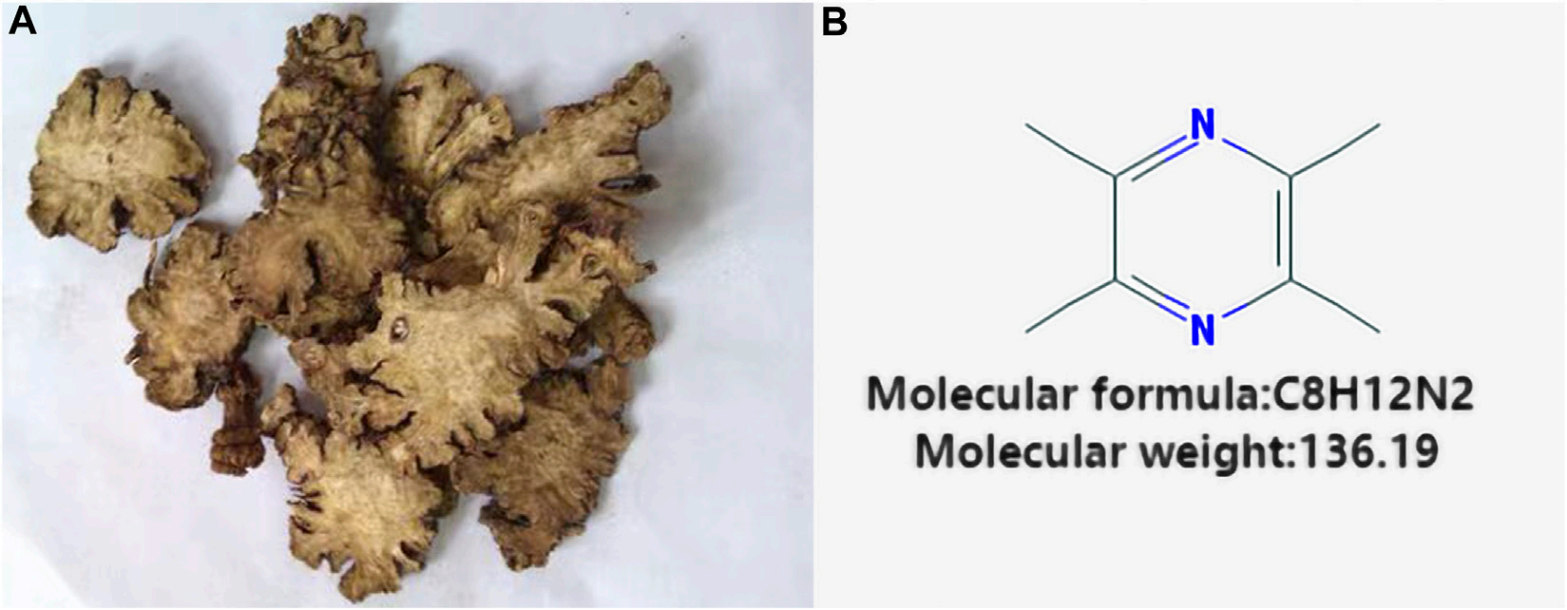
Vegetal products of *Ligusticum striatum DC* and chemical structure of Ligustrazine. **(A)** Vegetal products of *Ligusticum striatum DC*. **(B)** Chemical structure of Ligustrazine.

**FIGURE 2:**
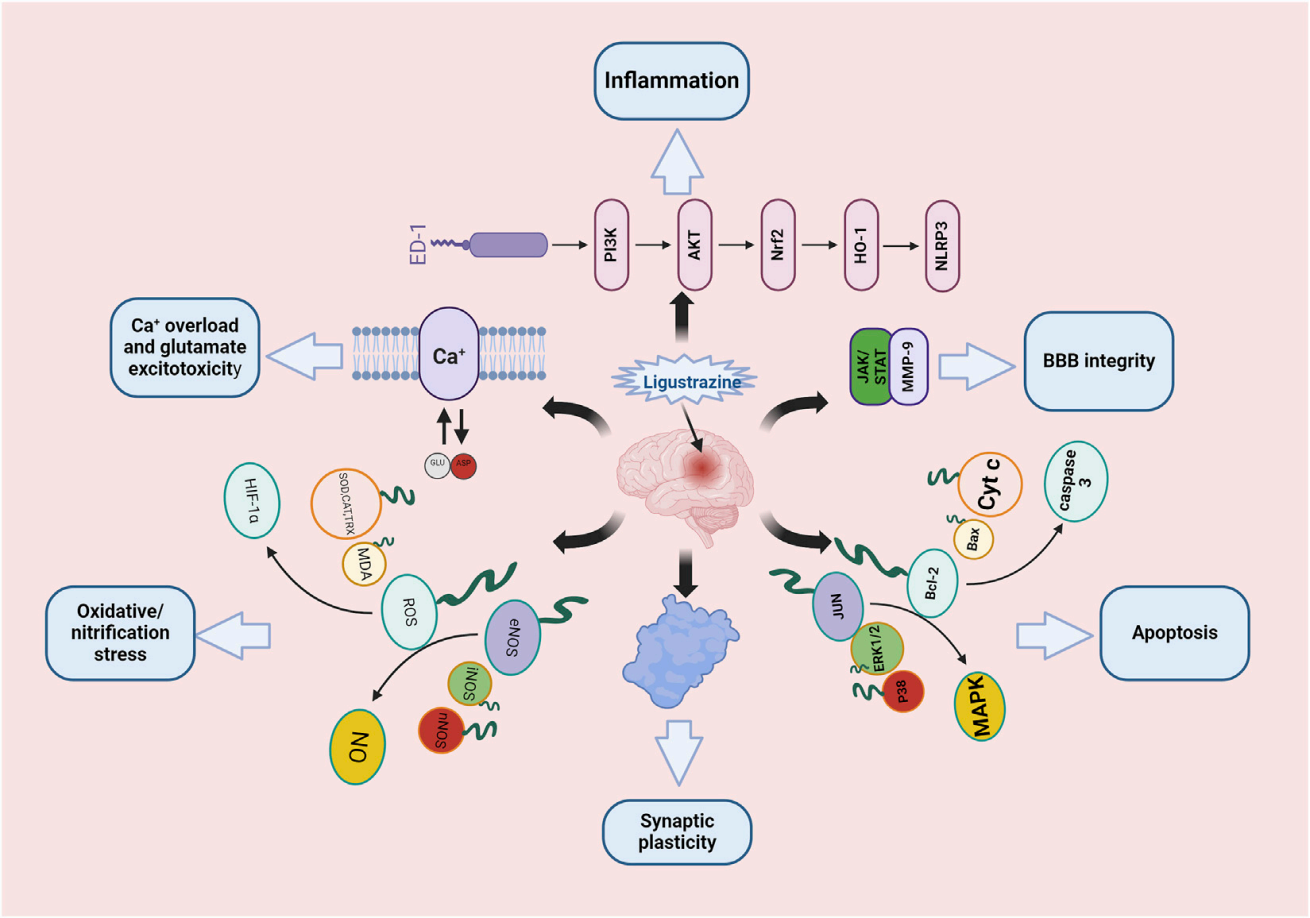
Mechanism diagram of ligustrazine intervention in CIS.

Ligustrazine, an alkaloid monomer derived from the dried rhizome of *Ligusticum striatum DC* [Apiaceae] (Figure 1). *Ligusticum striatum DC* was first documented in *Shennong’s Materia Medica*, a compilation of Chinese herbal medicine information dating back to 2800 BC (Li et al., 2021). According to traditional Chinese medicine, CIS is attributed to wind, fire, phlegm, blood stasis, and deficiency, with *L. striatum DC* to improve clinical symptoms of CIS by enhancing qi and blood circulation, activating blood flow, and resolving blood stasis. Modern scientific studies have shown that *L. striatum DC* can be effective in stroke treatment, with its mechanism of action linked to the regulation of the TNF/IL-17 signaling pathway (Zhang et al., 2023). Ligustrazine, an active compound in *L. striatum DC*, plays a key role in these therapeutic effects. Preclinical studies have demonstrated that ligustrazine has the potential to mitigate CIS injury through various mechanisms, including alleviating inflammatory response, resisting apoptosis, protecting the BBB, combating oxidative stress, inhibiting calcium overload and glutamate excitotoxicity, and enhancing synaptic plasticity (Figure 2). Specifically, ligustrazine has been shown to regulate inflammation-related factors by inhibiting the immunoreactivity of ED-1, DNA fragmentation, caspase-3 activation, and Cyt c release. Additionally, it inhibits the activation of the JAK/STAT signaling pathway and matrix metalloproteinase-9 (MMP-9) expression to reduce BBB permeability. Ligustrazine also reduces the concentration of ROS and malondialdehyde (MDA) while upregulating the activity of superoxide to relieve oxidative stress. Furthermore, it intervenes in the course of ischemia-reperfusion by reducing Ca2^+^ overload and trough amino acid excitotoxicity, as well as improving synaptic ultrastructure to enhance synaptic plasticity (Liu et al., 2022).

While numerous studies have delved into the neuroprotective effects and potential mechanisms of ligustrazine in CIS models, most have primarily focused on specific pathways or a limited set of efficacy indicators. A comprehensive and quantitative analysis of the various mechanisms of action of ligustrazine in CIS has yet to be reported. Although some studies have conducted systematic reviews and meta-analyses of clinical trials on ligustrazine for treating CIS (Shao et al., 2021), it remains essential to quantify and evaluate its mechanism of action through preclinical meta-analysis. This study aims to systematically evaluate and quantify the intervention effect of ligustrazine on CIS across multiple mechanisms and efficacy indicators. By synthesizing existing data, the study seeks to uncover the therapeutic potential and multifaceted action mechanism of ligustrazine in CIS, offering insights for future clinical research.

## 2 Materials and methods

### 2.1 Search strategy

Two researchers conducted independent searches on several databases including CNKI, Wanfang, VIP, CBM, Cochrane Library, PubMed, Web of Science, and Embase. The studies focused on the intervention of ligustrazine in animal models of CIS. The search period ranged from the establishment of the databases to January 2024. Additionally, the researchers traced the references of included articles and visited the International Clinical Trials Registry of the National Institutes of Health and the Chinese Clinical Trials Registry to supplement the acquisition of relevant literature. Any disagreements were resolved by involving a third researcher. The search terms used were: “brain infarction” or “brain ischemia” or “cerebral ischemia reperfusion injury” or “cerebral thrombosis” or “cerebral embolism” or “ischemic stroke” or “middle cerebral artery occlusion” or “MCAO” and “tetramethylpyrazine” or “ligustrazine".

### 2.2 Inclusion criteria and outcome indicators

#### 2.2.1 Outcome indicators

1) **Primary outcome measures:** Neurological deficit score (NDS).
2) **Secondary outcome measures**:
a. Percentage of infarct volume
b. Brain water content
c. Inflammation related factors, including tumor necrosis factor-α (TNF-α), interleukin -1β (IL-1β) and interleukin −6 (IL-6).
d. Oxidative stress-related indicators, including Superoxide Dismutase (SOD), Nitric Oxide Synthase (NOS), MDA and Nitric Oxide (NO).
e. Apoptosis marker caspase-3.
f. BBB -related protein Claudin-5.

#### 2.2.2 Inclusion criteria

1)Study Type: This study focuses on animal experiments conducted on rats or mice using permanent CIS or ischemic stroke/reperfusion models. 2) Interventional measures: experimental group was treated with ligustrazine without restriction, control group was treated with placebo only, placebo included saline, PBS, etc., 3) Language: There are no language restrictions for this study.

#### 2.2.3 Exclusion criteria

1) Animal experiments in permanent CIS or ischemic stroke/reperfusion models other than rats or mice. 2) Drug combination therapy. 3) Review, conference, case report, dissertation, clinical trial research. 4) Duplicated studies are excluded according to time. 5) Failure to reach the result index or to extract effective indicators. 6) Clinical studies or *in vitro* experiments.

### 2.3 Literature screening and data extraction

Two reviewers independently screened the literature, extracted data, and cross-checked. In the event of disagreements, a third party was consulted for assistance in making a judgment. If any data were missing, the authors were contacted to provide additional information. During the literature screening process, the title and abstract were initially read, and after excluding obviously irrelevant literature, the full text was further examined to determine its inclusion. The data extraction process mainly involved capturing the basic characteristics of the study, such as the study name, publication date, animal species, animal weight, and animal model. It also included specific details such as intervention measures, control measures, intervention time, drug dosage, and other relevant information used in the study. Key elements for evaluating the risk of bias were also considered, along with the outcome indicators involved in the study. For studies reporting data only in the form of images, we used the GetData Graph Digitizer software to extract the data from the images.

### 2.4 Quality evaluation of the included studies

In this study, two researchers conducted a systematic review of the included studies using the Cochrane risk of bias assessment tool (Cumpston et al., 2019). The evaluation focused on several key aspects, including selection bias (whether the random sequence generation was adequately described and whether allocation concealment was implemented), implementation bias (whether the subjects and experimenters were blinded), measurement bias (whether the outcome assessors were blinded), follow-up bias (whether the outcome data was complete), reporting bias, and other potential biases.

### 2.5 Statistical methods

Statistical analysis of the data was conducted using Review Manager 5.4 software, and sensitivity analysis was performed using Stata16. This study utilizes relative risk (RR) and 95% confidence interval (CI) as indicators for dichotomous variables, while Standardized mean difference (SMD) and 95% CI are used for continuous variables. Heterogeneity between studies was analyzed using the χ^2^ test. If I^2^ ≤ 50%, it indicates small heterogeneity between studies, and the fixed effects model is applied. On the other hand, if I^2^ ≥ 50%, it suggests large heterogeneity between studies, and a random effects model is utilized. Subgroup analysis or sensitivity analysis is employed when necessary. The presence of publication bias was assessed by analyzing outcome indicators from studies with a minimum of 10. A statistically significant difference is considered when *p* < 0.05.

### 2.6 Publication bias

Publication bias was assessed using a funnel plot and calculated with STATA 16 using Begg’s test.

## 3 Results

### 3.1 Literature screening process and results

**FIGURE 3:**
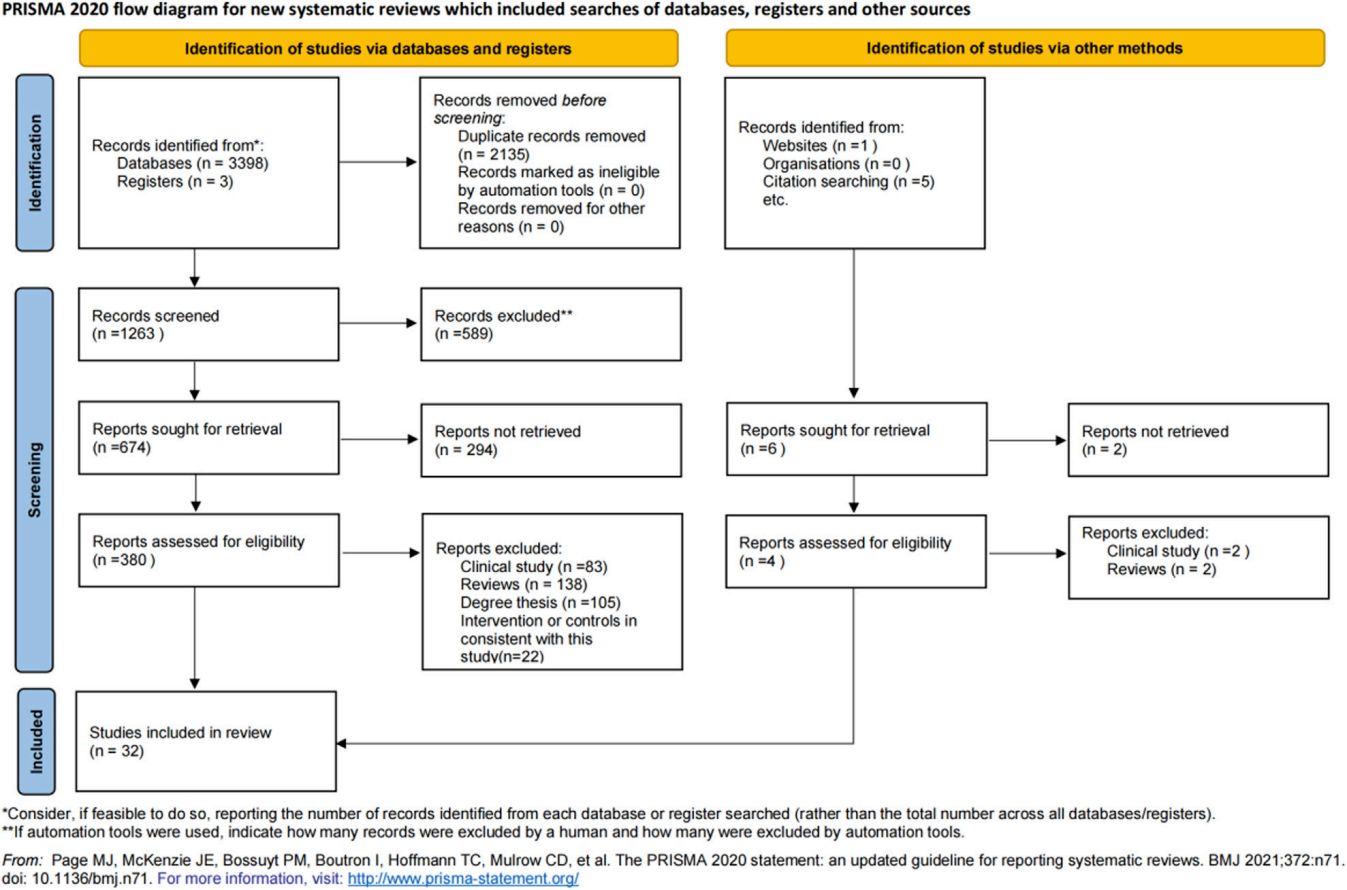
PRISMA flow chart for literature screening.

A total of 3,398 relevant documents were retrieved. After applying the inclusion and exclusion criteria, 32 studies (Liao et al., 2004; Kao et al., 2006; Chang et al., 2007; Li et al., 2008; Qi et al., 2008; Ren et al., 2008; Zhang et al., 2008; Jia et al., 2009; Zhu et al., 2009; Hu et al., 2010a; Hu et al., 2010b; Chen et al., 2010; Fang et al., 2010; Ma and Chen, 2010; Xiao et al., 2010; Yang and Ren, 2011; Zhang et al., 2011; Kao et al., 2013; Li et al., 2013; Han et al., 2014; Tian et al., 2014; Tan et al., 2015; Liu, 2016; Li et al., 2017; Gong et al., 2019; Zhu et al., 2020; Ge et al., 2021; Li et al., 2022; Liang et al., 2022; Chang et al., 2023; Feng et al., 2023; Gao et al., 2023) were included. The detailed literature search and screening process is illustrated in Figure 3.

### 3.2 Basic characteristics of included literature

A total of 32 studies were included in this study. Among these, three studies used Wistar rats (Chang et al., 2007; Li et al., 2008; Zhang et al., 2011) weighing 250–320 g, two studies used Kunming mice (Hu et al., 2010a; Hu et al., 2010b) weighing 30–40 g, and one study used C57BL/6 mice (Li et al., 2022), but the weight of the mice was not reported. The remaining 26 studies utilized SD rats weighing 220–350 g. Five studies employed the MCAO model, while 27 studies used the MCAO/reperfusion model. Intraperitoneal injection was used in 29 studies, tail vein injection in two studies (Ma and Chen, 2010; Zhu et al., 2020), and oral gavage in one study (Qi et al., 2008). The basic characteristics of the 32 included documents are presented in Table 1, and information on the preparations used in the study can be found in supplementary documents.

**TABLE 1:** Basic characteristics of included studies.

Characteristics of studies included in the meta-analysis								
Study ID	Animal characteristics	Animal model	Ligustrazine dosage (mg/kg)	Method of administration	Interventions		Course	Outcome
					T	S		
Chang (2007)	Male Wistar rats (250–300 g)	MCAO/reperfusion model	20	Intraperitoneal injection	Ligustrazine	Vehicle	20 min before animal modeling	d, k
Chang (2023)	Male SD rats	MCAO model	20	Intraperitoneal injection	Ligustrazine	Vehicle	Administration 30 min before and 60 min after animal modeling	b, e
Chen (2010)	Male SD rats (280–320 g)	MCAO/reperfusion model	NA	Intraperitoneal injection	Ligustrazine	Vehicle	NA	g, i
Fang (2010)	Male SD rats (280–320 g)	MCAO/reperfusion model	80	Intraperitoneal injection	Ligustrazine	Vehicle	After animal modeling	b
Feng (2023)	Male SD rats (300 ± 20 g)	MCAO model	20	Intraperitoneal injection	Ligustrazine	Vehicle	4 h after MCAO	f
Gao (2023)	Male SD rats (220–280 g)	MCAO/reperfusion model	50	Intraperitoneal injection	Ligustrazine	Vehicle	Injection immediately after ischemia reperfusion	a
Ge (2021)	Male SD rats (250–300 g)	MCAO/reperfusion model	50	Intraperitoneal injection	Ligustrazine	Vehicle	Injection 24 h after molding	d, f, g, i, j
Gong (2019)	SD rats (240–280 g)	MCAO/reperfusion model	40	Intraperitoneal injection	Ligustrazine	Vehicle	12 h after animal modeling	a, b, c
Han (2014)	Male SD rats (290 ± 10 g)	MCAO/reperfusion model	50	Intraperitoneal injection	Ligustrazine	Vehicle	Injection immediately after reperfusion	b
Hu (2010a)	Kunming mice (30–40 g)	MCAO/reperfusion model	40	Intraperitoneal injection	Ligustrazine	Vehicle	After 3 days of continuous injections	h, j
Hu (2010b)	Kunming mice (30–40 g)	MCAO/reperfusion model	40	Intraperitoneal injection	Ligustrazine	Vehicle	After 3 days of continuous injections	h, j
Jia (2009)	Male SD rats (300–350 g)	MCAO/reperfusion model	20	Intraperitoneal injection	Ligustrazine	Vehicle	Intervention administered 1, 2, 4 and 6 h after reperfusion	a
Kao (2013)	Male SD rats (300–350 g)	MCAO model	20	Intraperitoneal injection	Ligustrazine	Vehicle	Intervention 30 min before and 60 min after MCAO	a, b
Kao (2006)	Male SD rats (300–350 g)	MCAO model	40	Intraperitoneal injection	Ligustrazine	Vehicle	Intervention 30 min before and 60 min after MCAO	b
Li (2008)	Wistar rats (250–320 g)	MCAO/reperfusion model	50	Intraperitoneal injection	Ligustrazine	Vehicle	30 min after reperfusion	a
Li (2013)	Male SD rats (250–300 g)	MCAO/reperfusion model	40	Intraperitoneal injection	Ligustrazine	Vehicle	2 h after surgery administration intervention	l
Li (2017)	Male SD rats (280 ± 20 g)	MCAO/reperfusion model	20	Intraperitoneal injection	Ligustrazine	Vehicle	Post-reperfusion drug administration intervention	a, b, c, d, e, f
Li (2022)	Male C57BL/6	MCAO/reperfusion model	20	Intraperitoneal injection	Ligustrazine	Vehicle	15 min administration intervention	a, b, e
Liang (2022)	Male SD rats (200–250 g)	MCAO/reperfusion model	72	Intraperitoneal injection	Ligustrazine	Vehicle	7 days continuous administration intervention	a, k
Liao (2004)	Male SD rats (300–350 g)	MCAO/reperfusion model	40	Intraperitoneal injection	Ligustrazine	Vehicle	60 min before MCAO administration intervention	c
Liu (2016)	Male SD rats (240–270 g)	MCAO/reperfusion model	30	Intraperitoneal injection	Ligustrazine	Vehicle	Two days before molding, once a day. 0.5 h before molding, 4h and 8 h after molding	a, b, g, i
Ma (2010)	Male SD rats (325 ± 25 g)	MCAO/reperfusion model	120	Tail vein injection	Ligustrazine	Vehicle	10 min before modeling intervention	e
Qi (2008)	Male SD rats (250–300 g)	MCAO/reperfusion model	40	Oral gavage	Ligustrazine	Vehicle	After successful animal modeling	a
Ren (2008)	Male SD rats (280 ± 20 g)	MCAO/reperfusion model	40	Intraperitoneal injection	Ligustrazine	Vehicle	12 h before and 12 h after animal modeling	a
Tan (2015)	Male SD rats (250–270 g)	MCAO/reperfusion model	20	Intraperitoneal injection	Ligustrazine	Vehicle	15 min before animal modeling	a, b, c
Tian (2014)	Male SD rats (220–250 g)	MCAO/reperfusion model	30	Intraperitoneal injection	Ligustrazine	Vehicle	Once a day for 7 days	a
Xiao (2010)	Male SD rats (180–200 g)	MCAO model	40	Intraperitoneal injection	Ligustrazine	Vehicle	MCAO 2 h later	c
Yang (2011)	Male SD rats (280 ± 20 g)	MCAO/reperfusion model	35	Intraperitoneal injection	Ligustrazine	Vehicle	Before and 12 h after surgery	a
Zhang (2008)	Male SD rats (280 ± 20 g)	MCAO/reperfusion model	80	Intraperitoneal injection	Ligustrazine	Vehicle	Once daily for 3 days before surgery and daily after surgery	g, h
Zhang (2011)	Wistar (280–320 g)	MCAO/reperfusion model	10	Intraperitoneal injection	Ligustrazine	Vehicle	After successful animal modeling	a, g
Zhu (2009)	Male SD rats (300–350 g)	MCAO/reperfusion model	20	Intraperitoneal injection	Ligustrazine	Vehicle	1, 2, 4, 6 h after reperfusion	a
Zhu (2020)	Male SD rats (220–250 g)	MCAO/reperfusion model	10	Tail vein injection	Ligustrazine	Vehicle	30 min before animal modeling	c, d

T, treatment group; C, control group; NA, not available; a: NDS; b: percentage of infarct volume; c: brain water content; d: TNF - α; e: IL-1β: f: IL-6; g: SOD; h: NOS; i: MDA; j: NO; k: caspase-3; l: Claudin-5.

### 3.3 Risk of bias

**FIGURE 4:**
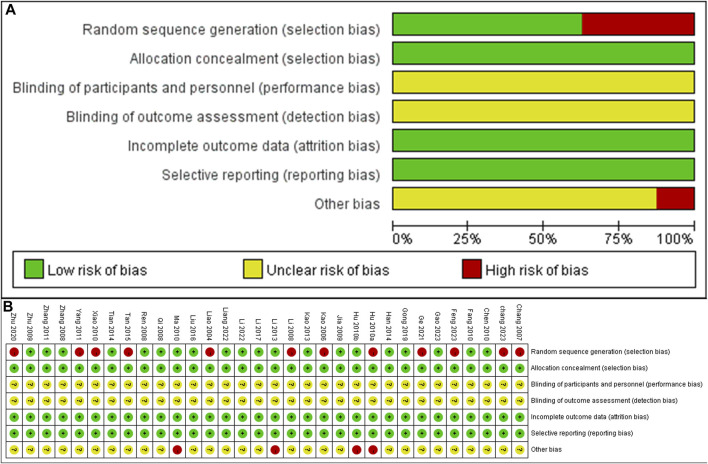
Risk of bias summary and Risk of bias graph **(A)** Risk of bias graph **(B)** Risk of bias summary.

A total of 12 studies did not provide a description of random random-sequence generation, indicating a high risk of selection bias. None of the studies mentioned allocation concealment, resulting in unclear judgments for both performance bias and detection bias. Since the animal’s baseline characteristics and data have been fully reported, both attrition bias and reporting bias are considered to have low risks. The authors of four studies (Hu et al., 2010a; Hu et al., 2010b; Ma and Chen, 2010; Li et al., 2013) had published similar papers during the same period, indicating a high risk. However, due to multiple factors influencing the risk of bias, the remaining 28 studies were judged to have an unclear risk. The specific bias situation is illustrated in Figure 4.

### 3.4 Analysis results

#### 3.4.1 NDS

**FIGURE 5:**
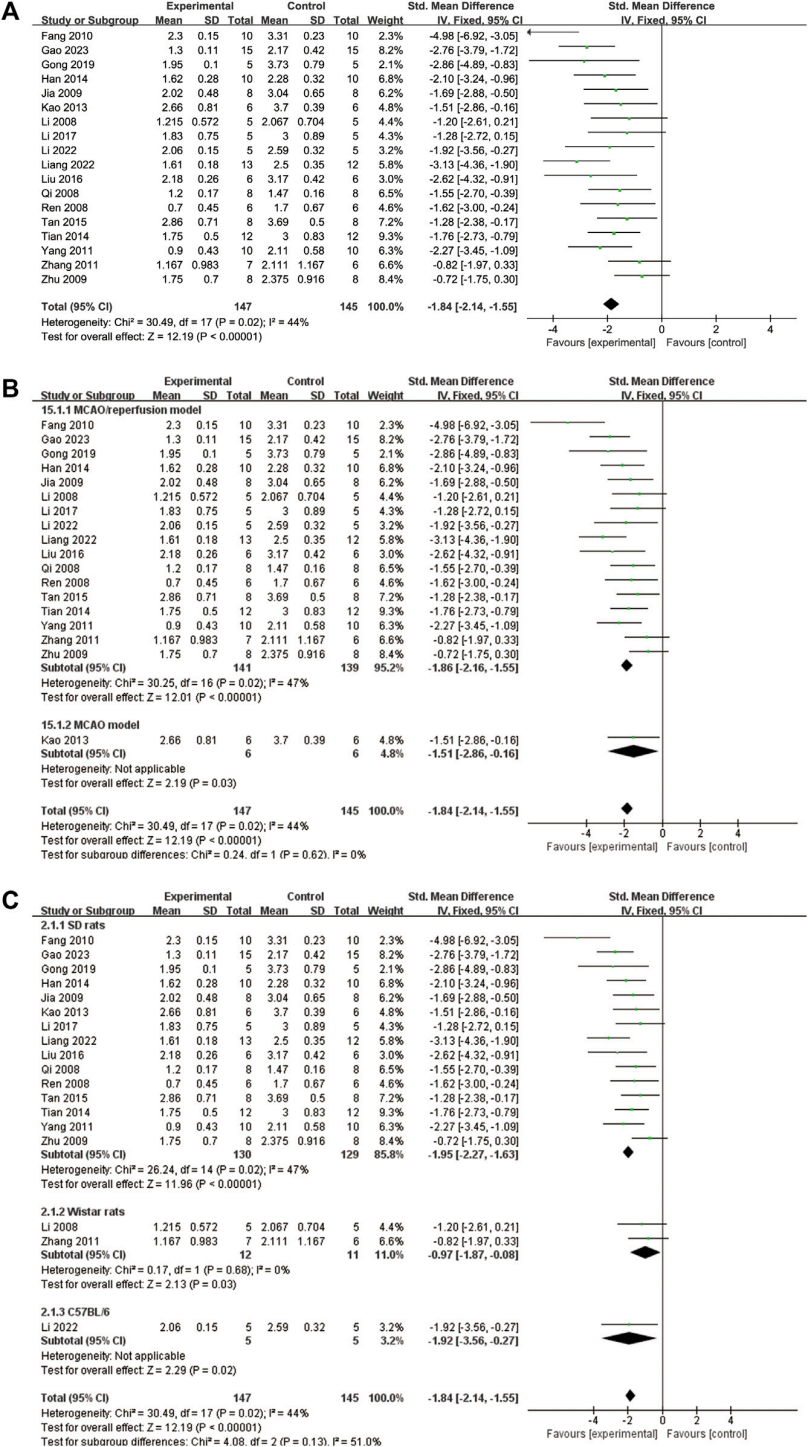
The forest plot of NDS. **(A)** The forest plot of NDS. **(B)** The forest plot of subgroup analysis based on the animal modeling method. **(C)** The forest plot of subgroup analysis based on the animal species.

A total of 18 studies (Li et al., 2008; Qi et al., 2008; Ren et al., 2008; Jia et al., 2009; Zhu et al., 2009; Fang et al., 2010; Yang and Ren, 2011; Zhang et al., 2011; Kao et al., 2013; Han et al., 2014; Tian et al., 2014; Tan et al., 2015; Liu, 2016; Li et al., 2017; Gong et al., 2019; Li et al., 2022; Liang et al., 2022; Gao et al., 2023) reported NDS. The heterogeneity test showed a significance of *p* = 0.02, with an I^2^ value of 44%. Therefore, a fixed effects model was adopted. The results indicated that ligustrazine could reduce the NDS after CIS (SMD = −1.84, 95% CI -2.14 to −1.55, *p* < 0.00001, Figure 5A). To enhance the reliability of the results, a subgroup analysis was conducted based on the animal modeling method and animal species, which demonstrated the robustness of the findings (Figures 5B, C).

#### 3.4.2 Percentage of infarct volume

**FIGURE 6:**
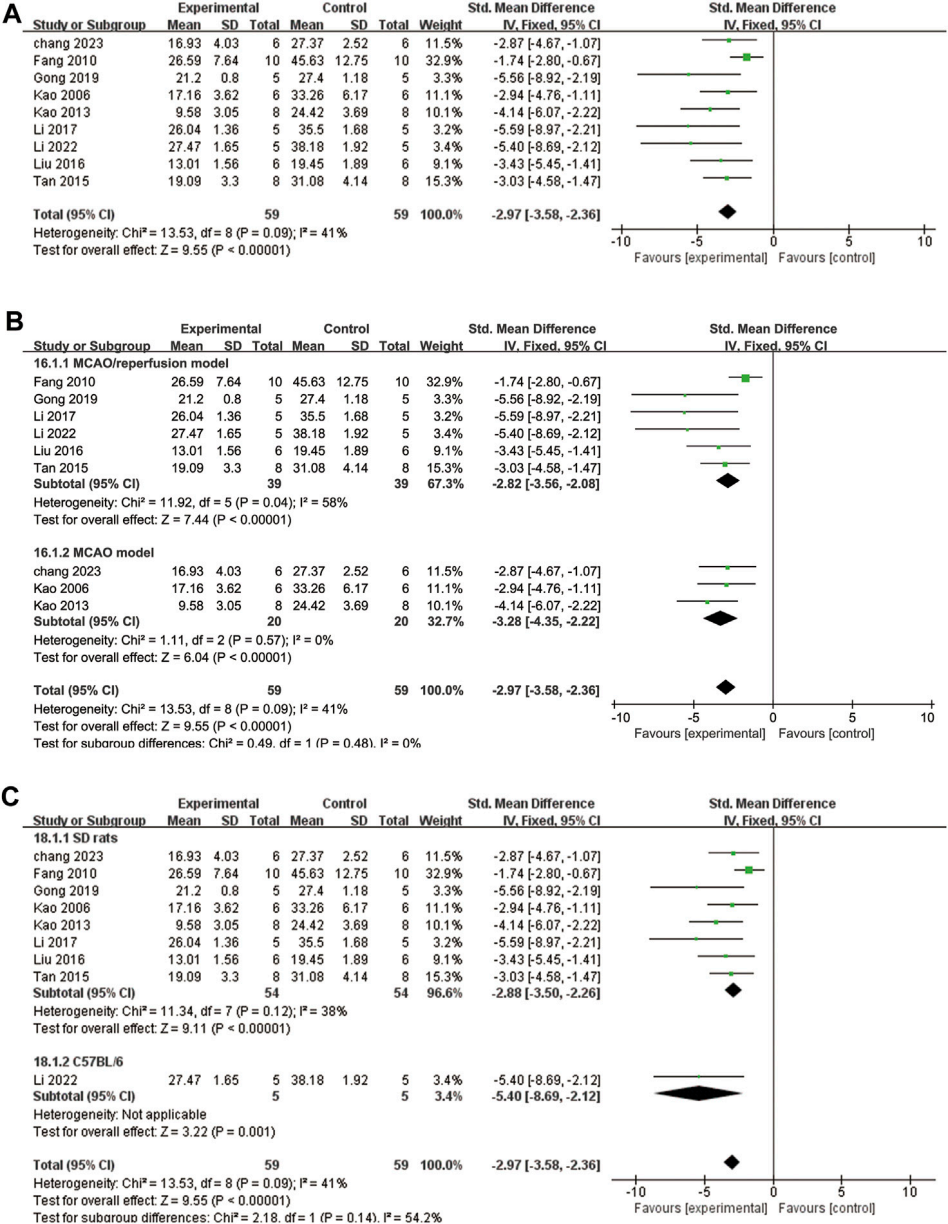
The forest plot of percentage of infarct volume. **(A)** The forest plot of percentage of infarct volume. **(B)** The forest plot of subgroup analysis based on the animal modeling method. **(C)** The forest plot of subgroup analysis based on the animal species.

A total of nine studies (Kao et al., 2006; Fang et al., 2010; Kao et al., 2013; Tan et al., 2015; Liu, 2016; Li et al., 2017; Gong et al., 2019; Li et al., 2022; Chang et al., 2023) reported the percentage of infarct volume. The heterogeneity test showed *p* = 0.09 and I^2^ = 41%. Therefore, a fixed effects model was adopted. The results demonstrated that ligustrazine significantly reduced the percentage of cerebral infarct volume in CIS (SMD = −2.97, 95% CI -3.58 to −2.36, *p* < 0.00001, Figure 6A). To enhance the reliability of the findings, subgroup analysis was conducted based on the animal modeling method and animal species, which confirmed the robustness of the results (Figures 6B, C).

#### 3.4.3 Brain water content

**FIGURE 7:**
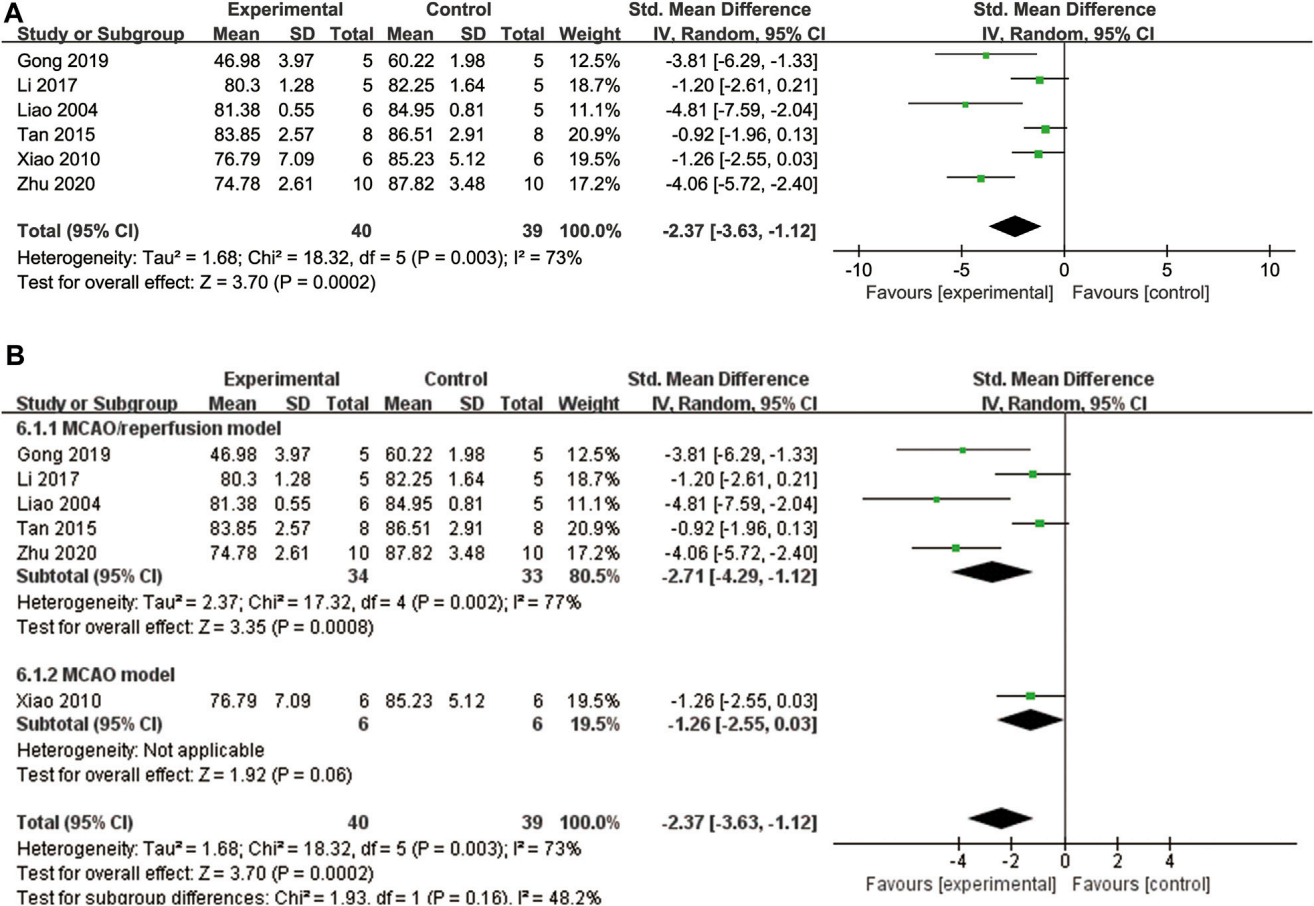
The forest plot of percentage of brain water content. **(A)** The forest plot of percentage of brain water content. **(B)** The forest plot of subgroup analysis based on the animal modeling method.

A total of six studies (Liao et al., 2004; Xiao et al., 2010; Tan et al., 2015; Li et al., 2017; Gong et al., 2019; Zhu et al., 2020) examined brain water content. To account for high heterogeneity (*p* = 0.003, I^2^ = 73%), a random effects model was applied. The findings indicate that ligustrazine can reduce brain water content in patients with CIS (SMD = −2.37, 95% CI -3.63 to −1.12, *p* = 0.0002, Figure 7A). To enhance the reliability of the results and identify the source of heterogeneity, we performed a subgroup analysis based on the animal modeling method. The results demonstrated the robustness of the findings (Figure 7B). However, the animal modeling method did not contribute to the observed heterogeneity according to the subgroup analysis (Figure 7B).

#### 3.4.4 Inflammation related factors

**FIGURE 8:**
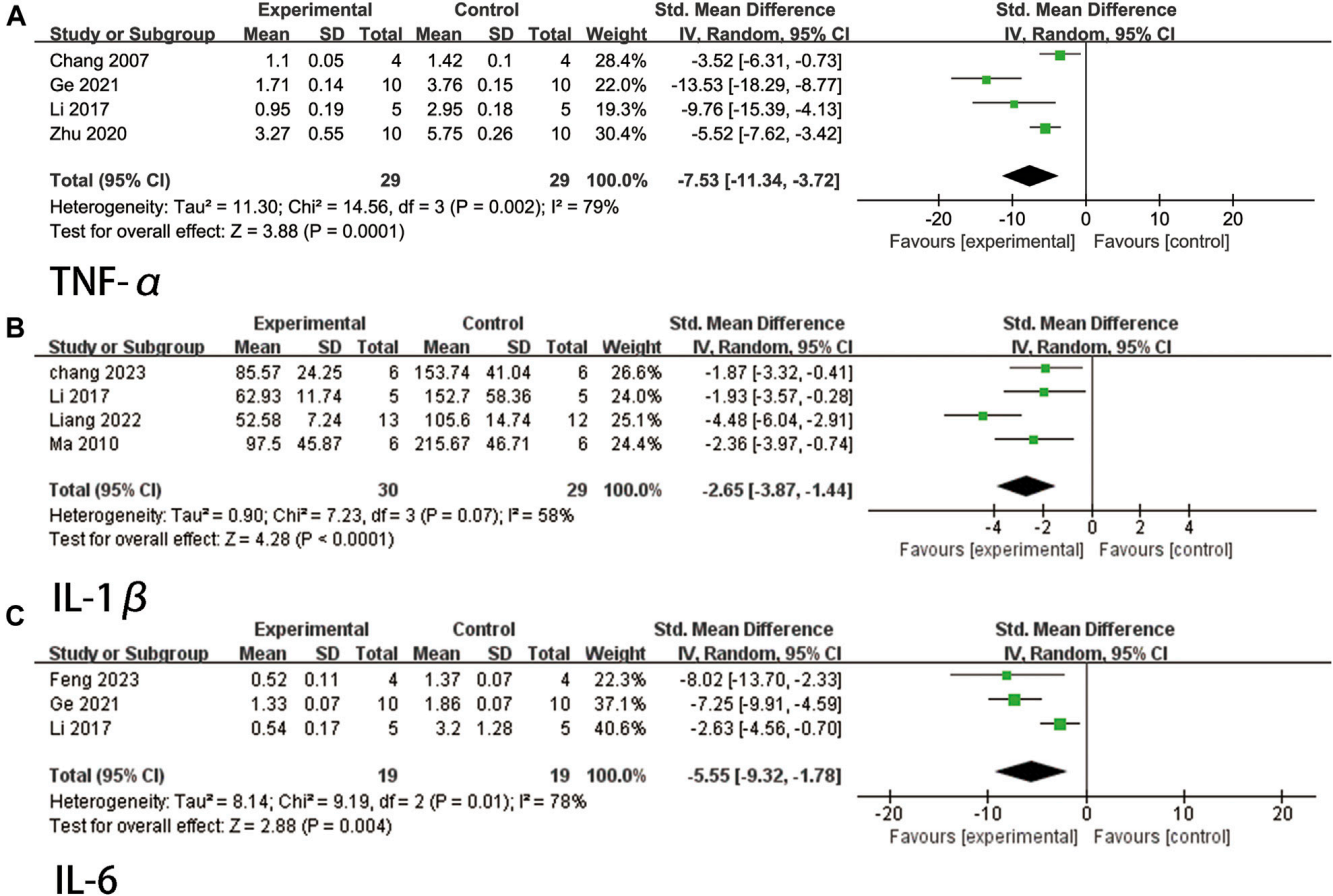
The forest plot of percentage of Inflammation related factors. **(A)** The forest plot of percentage of TNF-α. **(B)** The forest plot of percentage of IL-1β. **(C)** The forest plot of percentage of IL-6.

A total of four studies (Chang et al., 2007; Li et al., 2017; Zhu et al., 2020; Ge et al., 2021) reported TNF-α levels. Due to high heterogeneity (*p* = 0.002, I^2^ = 79%), a random effects model was used. The findings indicated that ligustrazine was effective in reducing TNF-α indicators in animals with CIS (SMD = −7.53, 95% CI -11.34 to −3.72, *p* = 0.0001, Figure 8A).

A total of four studies (Ma and Chen, 2010; Li et al., 2017; Liang et al., 2022; Chang et al., 2023) reported IL-1β levels. The heterogeneity test indicated a *p*-value of 0.07 and an I^2^ value of 58%. Therefore, a random effects model was employed. The findings demonstrated that ligustrazine had a significant impact in reducing IL-1β indicators in animals with CIS (SMD = −2.65, 95% CI -3.87 to −1.44, *p* < 0.0001, Figure 8B).

A total of three studies (Li et al., 2017; Ge et al., 2021; Feng et al., 2023) reported IL-6 levels. Due to high heterogeneity (*p* = 0.01, I^2^ = 78%), a random effects model was used. The findings indicated that ligustrazine could effectively reduce IL-6 indicators in animals with CIS (SMD = −5.55, 95% CI -9.32 to −1.78, *p* = 0.004, Figure 8C).

#### 3.4.5 Oxidative stress-related indicators

**FIGURE 9:**
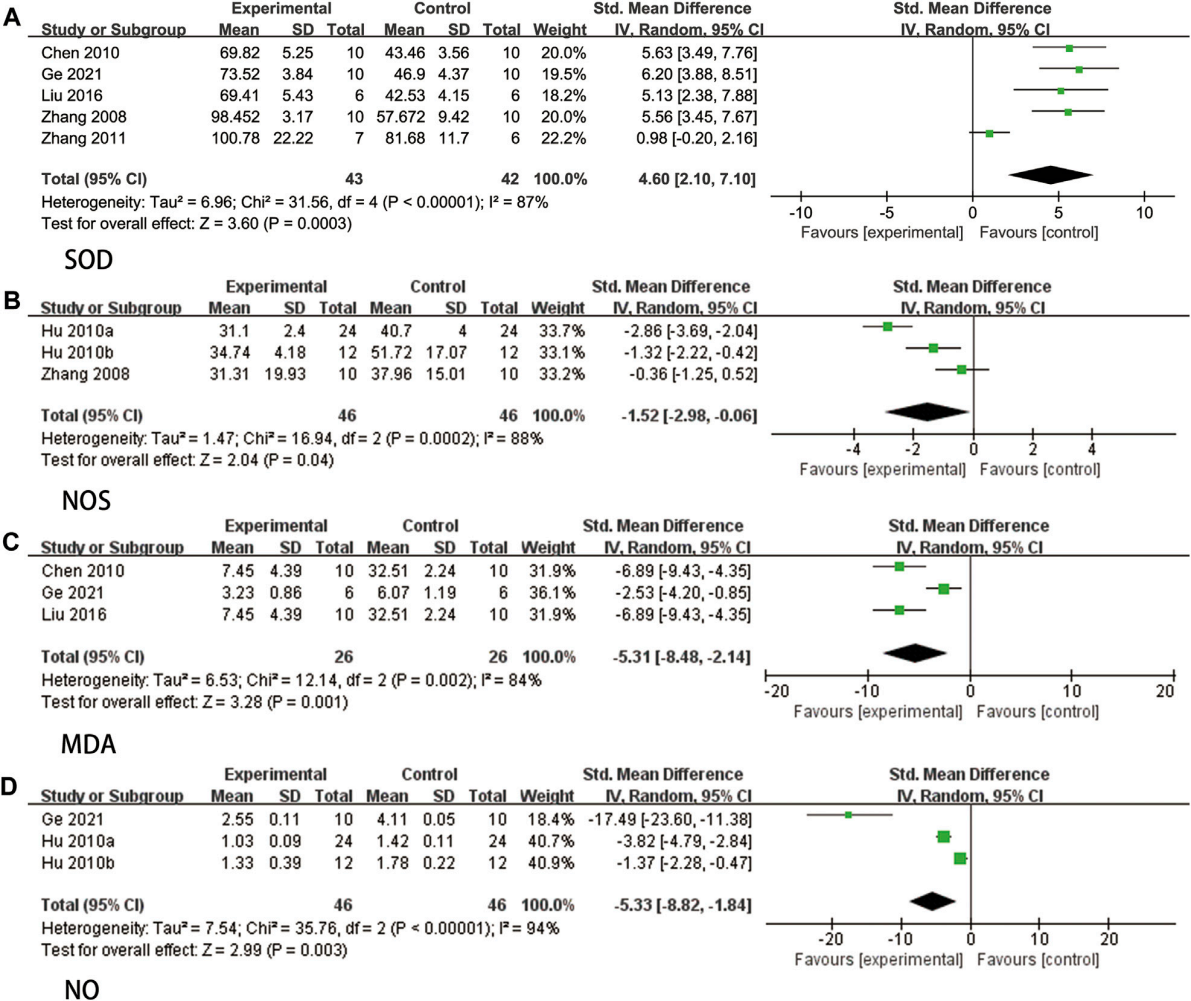
The forest plot of percentage of Oxidative stress-related indicators. **(A)** The forest plot of percentage of SOD. **(B)** The forest plot of percentage of NOS. **(C)** The forest plot of percentage of MDA. **(D)** The forest plot of percentage of NO.

Five studies (Zhang et al., 2008; Chen et al., 2010; Zhang et al., 2011; Liu, 2016; Ge et al., 2021) reported the measurement of SOD, and due to significant heterogeneity (*p* < 0.00001, I^2^ = 87%), a random effects model was employed. The findings demonstrated that ligustrazine had the ability to decrease SOD levels in animals with CIS (SMD = 4.60, 95% CI 2.10 to 7.10, *p* = 0.0003, Figure 9A).

Three studies (Zhang et al., 2008; Hu et al., 2010a; Hu et al., 2010b) reported NOS. Due to high heterogeneity (*p* = 0.0002, I^2^ = 88%), a random effects model was used. The results indicated that ligustrazine could reduce NOS indicators in animals with CIS (SMD = −1.52, 95% CI -2.98 to −0.06, *p* = 0.04, Figure 9B).

Three studies (Chen et al., 2010; Liu, 2016; Ge et al., 2021) reported MDA levels, and due to significant heterogeneity (*p* = 0.002, I^2^ = 84%), a random effects model was employed. The findings indicated that ligustrazine demonstrated a reduction in MDA indicators among animals with CIS (SMD = −5.31, 95% CI -8.48 to −2.14, *p* = 0.001, Figure 9C).

Three studies (Hu et al., 2010a; Hu et al., 2010b; Ge et al., 2021) reported NO, and due to high heterogeneity (*p* < 0.00001, I^2^ = 94%), a random effects model was employed. The findings indicated that ligustrazine had the potential to decrease NO indicators in animals with CIS (SMD = −5.33, 95% CI -8.82 to −1.84, *p* = 0.003, Figure 9D).

#### 3.4.6 Caspase-3

**FIGURE 10:**
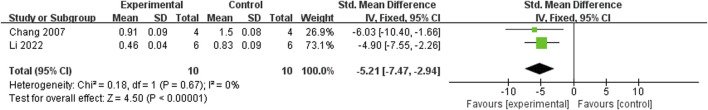
The forest plot of percentage of caspase-3.

Two studies (Chang et al., 2007; Li et al., 2022) reported caspase-3 levels, and the heterogeneity test showed no significant variation (*p* = 0.67, I^2^ = 0%). Therefore, a fixed effects model was used. The results indicated that ligustrazine had a significant effect in reducing caspase-3 levels in animals with CIS (SMD = −5.21, 95% CI -7.47 to −2.94, *p* < 0.00001, Figure 10).

#### 3.4.7 Claudin-5

**FIGURE 11:**
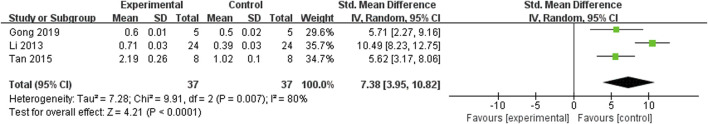
The forest plot of percentage of Claudin-5.

Three studies (Li et al., 2013; Tan et al., 2015; Gong et al., 2019) examined Claudin-5, and after conducting a heterogeneity test (*p* = 0.007, I^2^ = 80%), a random effects model was applied. The findings revealed that ligustrazine had the potential to decrease the Claudin-5 index in animals with CIS (SMD = 7.38, 95% CI 3.95 to 10.82, *p* < 0.0001, Figure 11).

### 3.5 Sensitivity analysis

#### 3.5.1 Brain water content

Due to the significant heterogeneity observed in this study, a sensitivity analysis was conducted. After excluding the study conducted by Zhu 2020 (Zhu et al., 2020), the heterogeneity decreased to 61%. These findings continue to support the notion that ligustrazine can effectively reduce the water content of brain tissue in animals with CIS. Further literature analysis revealed that only Zhu 2020 administered the treatment via tail vein injection, whereas the remaining studies utilized intraperitoneal injection. This observed heterogeneity is likely attributed to the differences in the method of administration.

#### 3.5.2 Inflammation related factors

TNF-α exhibits significant heterogeneity, prompting a sensitive analysis. Upon excluding Ge 2021 (Ge et al., 2021), the heterogeneity decreased to 50%. A comprehensive literature analysis identified a total of four studies contributing to this indicator. Notably, Ge 2021 utilized a dosage of 50 mg/kg, while the remaining three studies employed dosages of 20, 20, and 10 mg/kg respectively. Hence, it is speculated that the heterogeneity is attributable to the varying dosages of Ge 2021, resulting in divergent medication effects. As for IL-6, which also displays substantial heterogeneity, a heterogeneity analysis was conducted. After excluding Li 2017 (Li et al., 2017), the heterogeneity decreased to 0%. Literature analysis suggests that this heterogeneity may arise from differences in administration time and two other studies.

#### 3.5.3 Oxidative stress-related indicators

SOD exhibited high heterogeneity, thus a sensitivity analysis was conducted. After excluding Zhang 2011 (Zhang et al., 2011), the heterogeneity decreased to 0%. Literature analysis revealed that this study employed Wistar rats, while the other four studies utilized SD rats. Hence, the heterogeneity of SOD indicators may be attributed to the different rat species.

NOS also demonstrated high heterogeneity, thus a sensitivity analysis was conducted. After excluding Hu 2020a (Hu et al., 2010a), the heterogeneity decreased to 55%. Since only three studies were included, it is speculated that the heterogeneity is linked to the limited number of experimental animals. However, the research results still support the notion that ligustrazine can regulate oxidative stress through NOS indicators, thereby protecting animals with CIS.

MDA displayed high heterogeneity, prompting a sensitivity analysis, which resulted in a heterogeneity decrease to 0% after excluding Ge 2021 (Ge et al., 2021). Literature analysis suggested that the heterogeneity may be due to varying dosages.

The heterogeneity of NO is also high, thus a sensitivity analysis was performed. Despite excluding studies one by one, the heterogeneity remained high. It is speculated that this is due to the small number of experimental animals. Nevertheless, the research results still support the idea that ligustrazine can regulate oxidative stress through NO indicators, effectively protecting animals with CIS.

#### 3.5.4 Claudin-5

Claudin-5 exhibits high heterogeneity, thus necessitating a sensitivity analysis. Upon excluding the study conducted by Li 2013 (Li et al., 2013), the heterogeneity decreased to 0%. Further examination of the literature revealed that this reduction could be attributed to the limited sample size of experimental animals. Nevertheless, the research findings continue to support the notion that Ligustrazine can safeguard animals with CIS by modulating oxidative stress through Claudin-5 indicators.

### 3.6 Publication bias

**FIGURE 12:**
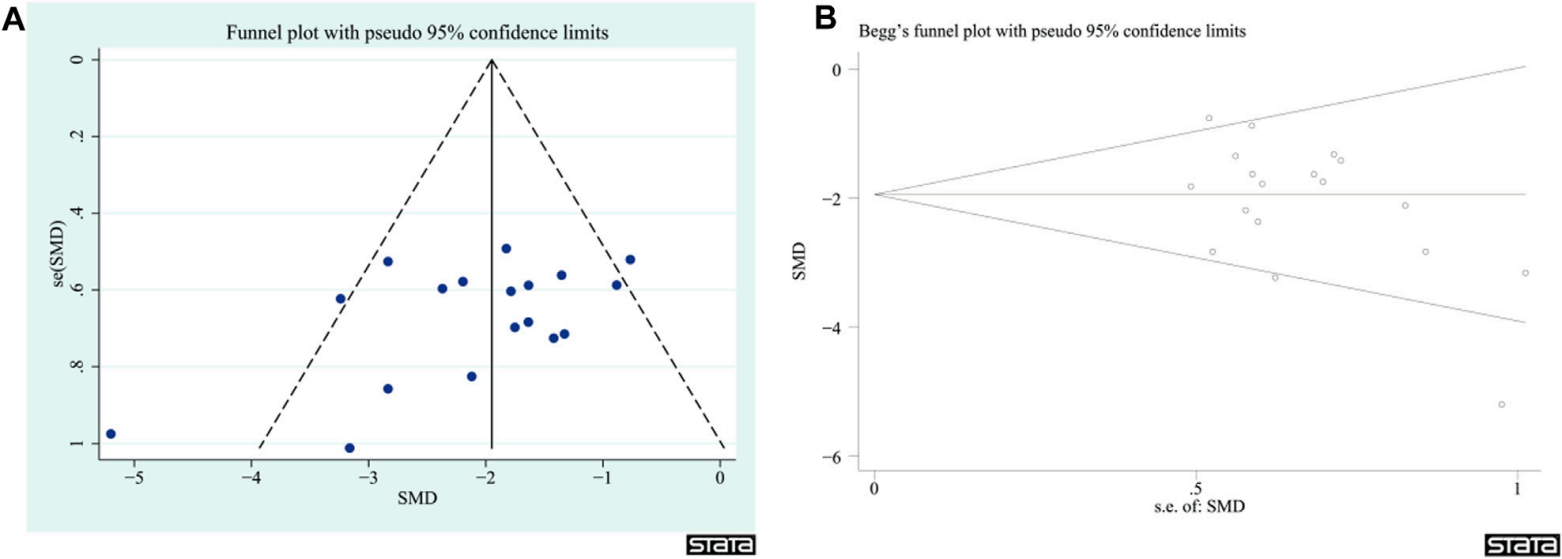
Publication bias plots. **(A)** Funnel plot of the NDS. **(B)** Begg’s plot of the NDS.

Given that the NDS had more than 10 articles among the outcome indicators, publication bias was evaluated. The findings indicated that there was no significant publication bias in the NDS (z = −1.63, *p* = 0.103, Figure 12); however, it is important to exercise caution when interpreting these results.

## 4 Discussion

CIS is a clinical syndrome caused by cerebral vascular disease, resulting in a disruption of cerebral blood supply, tissue necrosis due to brain tissue hypoxia and glucose deficiency, and neurological impairment (Chen et al., 2021). Current guidelines recommend thrombolysis and endovascular therapy (Kapil et al., 2017). However, thrombolytic treatment can result in rapid reoxygenation during reperfusion, leading to the generation of reactive oxygen species and subsequent oxidative stress reaction and inflammatory storm, which can be detrimental to the brain. Moreover, due to strict time constraints and various contraindications, many patients are unable to benefit from this treatment. As a result, complementary and alternative therapies are gaining increasing attention from clinical practitioners. Ligustrazine, a new calcium channel antagonist, has shown promise in the treatment of ischemic cerebrovascular disease and coronary atherosclerotic disease (Zhao et al., 2016).

Over the past 2 decades, there has been a growing body of evidence demonstrating the positive effects of ligustrazine on CIS in both *in vivo* and *in vitro* studies (Zhu et al., 2021). The mechanism of action of ligustrazine involves several beneficial processes, such as activating free radical scavenging (Yang et al., 2017; Zhang et al., 2018a), reducing BBB destruction (Tan et al., 2015; Gong et al., 2019), inhibiting inflammation (Zhou et al., 2019; Chang et al., 2023), maintaining mitochondrial function (Zhang et al., 2018b), preventing apoptosis (Chang et al., 2007), and promoting oligodendrogenesis and gliogenesis (Zhang et al., 2018a). To date, no studies have conducted a systematic review and meta-analysis on the mechanism of action of ligustrazine. In this study, we aimed to provide a comprehensive evaluation of various studies on the treatment of CIS using ligustrazine. Ultimately, we included 32 studies to elucidate the effectiveness and mechanism of action of this treatment. Studies have demonstrated that following an ischemic stroke, there is a likelihood of significant brain edema and the occurrence of large-area infarcts. Additionally, neurons may undergo extensive necrosis and apoptosis, leading to severe damage to the cell structure (Jurcau and Simion, 2021; Fu et al., 2023). This study conducted a statistical analysis of the included studies and found that ligustrazine exhibits a significant improvement in cerebral edema, reduction in cerebral infarction area, and amelioration of neurological damage. Furthermore, the statistical analysis of outcome indicators in the included studies revealed that ligustrazine can effectively enhance inflammatory factor-related indicators (TNF-α, IL-1β, and IL-6), oxidative stress-related indicators (SOD, NOS, MDA, and NO), apoptosis indicator caspase-3, and BBB permeability-related protein Claudin-5.

Inflammation plays a crucial role in the pathophysiology of ischemic stroke. Specifically, inflammatory cytokines IL -1, IL -6, and TNF have been identified as key regulators of the immune response following ischemic stroke (Lambertsen et al., 2019; Clausen et al., 2020). In the hyperacute window after ischemia, resident microglia are recruited from the site of cellular injury due to the action of various cytokines such as TNF-α, IL-6, and IL-1β. This localized inflammation then triggers systemic inflammation, resulting in the breakdown of the BBB, brain edema, and neuronal death (Jin et al., 2010; Naik et al., 2023). The findings of this study demonstrate that ligustrazine can effectively modulate TNF - α, IL-6, and IL-1β levels in animals with ischemic stroke, thereby improving the damage caused by the stroke.

During ischemia reperfusion, the level of NO initially increased, then decreased, and then increased again. In pathological conditions, there was a significant increase in inducible NOS (INOS), which led to excessive production of NO. This excessive NO production caused damage to nerve tissue, resulting in a neurotoxic effect (Fan et al., 2022) Cerebral ischemia reperfusion injury leads to the production of a large amount of oxygen free radicals, which in turn cause peroxidation reactions and changes in the basic characteristics of cell membranes. This process results in the production of MDA in large quantities. SOD, as a main enzyme responsible for scavenging oxygen free radicals, plays a crucial role in protecting cells against oxidative damage (Korenić et al., 2014; Deng et al., 2022). In this study, mice in the ligustrazine intervention group showed a significant decrease in oxidative stress index levels of NO, NOS, and MDA, as well as a significant increase in the antioxidant index SOD level. These findings suggest that ligustrazine effectively regulates the oxidative balance in brain tissue injury, thereby reducing damage caused by oxygen free radicals and protecting nerve cells.

Apoptosis, a process of programmed cell death, is activated during cerebral ischemia (Love, 2003). Caspase-3, a member of the cysteine protease family, plays a crucial role in mediating apoptosis (Uzdensky, 2019). The destruction of the BBB is a significant pathophysiological process in acute ischemic stroke, which can result in destructive malignant brain edema and hemorrhagic transformation (Qiu et al., 2021). Previous research has demonstrated that ligustrazine has the ability to penetrate the BBB and distribute across various brain regions (Tsai and Liang, 2001). However, its biological half-life when taken orally is limited to 0.5–2 h. Utilizing ligustrazine - loaded liposomes (Xia et al., 2016), transdermal administration drugs (Qi et al., 2002), intranasal administration (Feng et al., 2009), and intraocular administration (Mao et al., 2019) can enhance the efficiency of ligustrazine in crossing the BBB and reaching the brain. Moreover, ligustrazine exhibits protective effects against CIS damage by reducing BBB permeability (Tan et al., 2015; Jin et al., 2021), possibly through the inhibition of the JAK/STAT signaling pathway (Gong et al., 2019) and the reduction of MMP-9 levels (Jin et al., 2021). Claudin-5, a specific protein found in the tight junctions of brain microvascular endothelial cells, has been identified as an inducer of BBB formation (Ueno, 2007). The analysis conducted in this study reveals that ligustrazine can reduce the expression of Caspase-3 and increase Claudin-5 levels. These findings suggest that ligustrazine may protect against cerebral ischemic injury by reducing apoptosis and repairing the BBB.

We conducted a meta-analysis of animal studies investigating the effects of ligustrazine intervention in CIS for the first time. We summarized and classified the indicators included in the study, finding that the mechanism of ligustrazine is closely related to inflammatory response, oxidative stress, apoptosis, and BBB permeability. This provides valuable insights for future studies in this area. However, there are several limitations in our study that should be acknowledged. Firstly, most of the included articles did not mention blinding of participants and results, which increases the risk of bias. Secondly, the secondary outcome indicators in our study showed heterogeneity. Although we identified the source of heterogeneity through sensitivity analysis, the limited data available may have led to inaccurate results. Thirdly, some indicators had small sample sizes, which increases the risk of bias. Fourthly, the safety of ligustrazine could not be evaluated in our study as most included studies did not record adverse reactions in animal models. Lastly, the extraction of outcome measures from images using GetData Graph Digitizer software may introduce inaccuracies. These limitations should be considered when interpreting the findings of our study.

A meta-analysis of clinical trials examining the combination of ligustrazine injection with western medicine for acute cerebral infarction showed better efficacy when compared to using western medicine alone (Shao et al., 2021). These findings align with the results of our study. While ligustrazine may cause damage to the gastrointestinal, peripheral nervous, or central nervous systems, most adverse effects are self-limiting and the compound is generally considered safe (Lin et al., 2022). However, due to the limited depth of research on the toxicology and side effects of ligustrazine, it is crucial to strictly adhere to the recommended indications and consider the patient’s drug allergy history. Researchers should prioritize investigating the toxicology and side effects of ligustrazine in patients with CIS to enhance its therapeutic potential for human use.

## 5 Conclusion

Our study suggests that ligustrazine has a protective effect on animal models of CIS. The mechanism behind this effect may be attributed to the reduction of inflammation and oxidative stress, inhibition of apoptosis, and repair of BBB permeability. However, discrepancies between animal models of CIS and the physiological and pathological processes in humans pose challenges in translating preclinical findings to clinical applications. Future preclinical investigations on ligustrazine for CIS treatment should adhere rigorously to predefined protocols to mitigate bias in subsequent studies.

## Data Availability

The original contributions presented in the study are included in the article/Supplementary material, further inquiries can be directed to the corresponding author.

## Abbreviations

APC, 3K3A-activating protein C; BBB, blood-brain barrier; CBM, Chinese Biomedical Literature Database; CI, confidence interval; CIS, cerebral ischemic stroke; CNKI, China National Knowledge Infrastructure; HPLC, high-performance liquid chromatography; HUK, human urinary kininogenase; IL, interleukin; INOS, inducible NOS; MCAO, middle cerebral artery occlusion; MDA, malondialdehyde; MMP-9, matrix metalloproteinase-9; NDS, neurological deficit score; NO, nitric oxide; NOS, nitric oxide synthase; RR, relative risk; SMD, standardized mean difference; SOD, superoxide dismutase; TNF, tumor necrosis facto; tPA, tissue plasminogen activator; VIP, VIP Database.
